# Genomic and neoantigen evolution from primary tumor to first metastases in head and neck squamous cell carcinoma

**DOI:** 10.18632/oncotarget.27907

**Published:** 2021-03-16

**Authors:** Charles R. Schutt, Hua Sun, Jaya Sarin Pradhan, Yvonne Saenger, Jessica Ley, Douglas Adkins, Matthew Ingham, Li Ding, Brian A. Van Tine

**Affiliations:** ^1^Division of Medical Oncology, Washington University in St. Louis, St. Louis, MO, USA; ^2^McDonnell Genome Institute, Washington University in St. Louis, St. Louis, MO, USA; ^3^Department of Pathology and Cell Biology, Columbia University, New York, NY, USA; ^4^Division of Hematology/Oncology, Columbia University, New York, NY, USA; ^5^Department of Genetics, Washington University in St. Louis, St. Louis, MO, USA; ^6^Division of Pediatric Hematology and Oncology, St. Louis Children’s Hospital, St. Louis, MO, USA; ^7^Siteman Cancer Center, St. Louis, MO, USA; ^*^Co-first authors

**Keywords:** head and neck squamous cell carcinoma, neoantigens, mutational evolution, tumor relapse, immune cell infiltration

## Abstract

Head and neck cell squamous-cell carcinomas (HNSCC) are a group of common cancers typically associated with tobacco use and human papilloma virus infection. Up to half of all cases will suffer a recurrence after primary treatment. As such, new therapies are needed, including therapies which promote the anti-tumor immune response. Prior work has characterized changes in the mutation burden between primary and recurrent tumors; however, little work has characterized the changes in neoantigen evolution. We characterized genomic and neoantigen changes between 23 paired primary and recurrent HNSCC tumors. Twenty-three biopsies from patients originally diagnosed with locally advanced disease were identified from the Washington University tumor bank. Whole exosome sequencing, RNA-seq, and immunohistochemistry was performed on the primary and recurrent tumors. Within these tumors, we identified 6 genes which have predicted neoantigens in 4 or more patients. Interestingly, patients with neoantigens in these shared genes had increased CD3+ CD8+ T cell infiltration and duration of survival with disease. Within HNSCC tumors examined here, there are neoantigens in shared genes by a subset of patients. The presence of neoantigens in these shared genes may promote an anti-tumor immune response which controls tumor progression.

## INTRODUCTION

Head and neck cancer are a group of heterogeneous tumors with an estimated 644,000 new cases per year worldwide [1]. Head and neck cancers represent 3% of all new cancer cases and 2% of all cancer-related deaths. Most head and neck cancers have squamous-cell carcinoma morphology (HNSCC). Risk factors for HNSCC include tobacco use and human papilloma virus (HPV) infection [2]. In locally advanced disease, current therapies include combinations of resection, radiotherapy, and chemotherapy [3]. Although these treatments may lead to cure, relapse of disease occurs in 30–50% of patients [4, 5].

Current therapies for relapsed or metastatic HNSCC include immunotherapy, chemotherapy, or cetuximab [6]. Immunotherapy was shown to prolong overall survival in comparison to chemotherapy given with or without cetuximab [7–10]. A relatively new FDA-approved type of therapy for HNSCC are immune checkpoint inhibitors. Immune checkpoint molecules, such as PD-1, PD-L1, and CTLA-4, are surface molecules on the surface of activated immune cells [11]. The binding of PD-1 to PD-L1 or CTLA-4 to CD80/CD86 inhibits the immune response. By blocking the binding of these checkpoint molecules, the immune response against the tumor is licensed to continue.

The efficacy of checkpoint inhibitors in HNSCC indicates a role for the immune system in the control and elimination of this disease. The infiltration of immune cells, including T cells, into tumor is associated with improved outcomes and longer survival in HNSCC [12–17]. The infiltrating T cells release granules containing perforin and granzyme A and B which directly kill tumor cells or release other cytokines and chemokines that promote the anti-tumor immune response and alter the tumor microenvironment [18]. For example, infiltrating T cells release interferon gamma which increases expression of PD-L1 and CTLA-4, which may increase the efficacy of immune checkpoint therapy [19, 20].

During the progression of cancer, there is an increased mutational burden. These mutations can result in the development of neoantigens. These neoantigens may mark some clones for immunoediting and elimination. However, the clones which escape immunoediting are the source of cancer cell persistence, relapse, and metastasis. Multiple studies have characterized changes in mutation burden in HNSCC [21–24], when comparing primary and metastatic tumors, no studies have characterized the shifting neoantigen burden between primary and metastatic tumors within HNSCC. In this study, we characterized the mutational and neoantigen burden between primary and first recurrence tumors in 23 patients with HNSCC. In this analysis, primary and recurrent tumors were identified that had neoantigens in shared genes in multiple patients. These patients had increased CD8+ cell infiltration and increased expression of cytolytic gene expression. This study provides the justification for looking at a larger dataset in a prospective manner for the identification of recurrent neoantigens in the evolution of HNSCC.

## RESULTS

### Patient demographics

Twenty-three HNSCC patients were identified that were consented to the Washington University tumor bank and had genetic material available for germline, primary tumor and first recurrence/metastases (patient characteristics are listed in Table 1). Males represented 17 of 23 patients (74%) of the patient population. Tobacco smokers represented 14/23 (61%), 7 (30%) patients were non-smokers, 1 (4%) patient chewed tobacco and 1 (4%) patient smoked marijuana. Primary tumors are in the oral cavity (9/23, 39%), oropharynx (7/23, 30%), larynx (6/23, 26%), and hypopharynx (1/23, 4%). All seven oropharyngeal patients (7/23, 30%) were positive for the human papilloma virus (HPV+). All of these percentages are similar to those reported in the cancer genome atlas [25]. In our patient population, 20 of the 23 patients (87%) received radiation, 13 of 23 (57%) received cisplatin, 7 of the 23 patients (30%) received no chemotherapy, and 3 of 23 patients (13%) received Cetuximab (Table 2).

**Table 1:** Patient demographics

Anatomic site	P16_status	Smoking History	Gender	Stage	Patient ID
Oropharynx	Positive	15 Pack-years	Male	Stage IVB T4bN2cM0	001
Oropharynx	Positive	Marijuana 1 Pack-years	Male	Stage IVA T2N2bM0	007
Oropharynx	Positive	Non-smoker	Male	Stage IVA T2N2bM0	008
Oropharynx	Positive	45 Pack-years	Male	Stage IVA T4aN2bM0	014
Oropharynx	Positive	10 Pack-years	Male	Stage IVA T1N2bM0	017
Oropharynx	Positive	Non-smoker	Male	Stage IVA T3N2bM0	018
Oropharynx	Positive	Non-smoker	Male	Stage IVA T2N2bM0	021
Hypopharynx	Negative	45 Pack-years	Male	Stage IVA T2N2aM0	005
Larynx	Negative	70 Pack-years	Male	Stage IVA T3N2M0	003
Larynx	Negative	14 Pack-years	Male	Stage IVA T3N3M0	004
Larynx	Negative	Chew for 42 years	Male	Stage I T1N0M0	009
Larynx	Negative	50 Pack-years	Female	Stage IVA T3N2bM0	011
Larynx	Negative	33 Pack-years	Female	Stage III T3N1M0	019
Larynx	Negative	68 Pack-years	Male	Stage III T2N1M0	013
Oral cavity	Negative	38 Pack-years	Male	Stage IVA T3N2M0	002
Oral cavity	Negative	Non-smoker	Male	Stage IVA T2N2bM0	010
Oral cavity	Negative	70 Pack-years	Male	Stage IVA T4N2bM0	012
Oral cavity	Negative	50 Pack-years	Male	Stage III T3N1M0	016
Oral cavity	Negative	50 Pack-years	Male	Stage IVA T4N2cM0	020
Oral cavity	Negative	Non-smoker	Male	Stage III T2N1M0	024
Oral cavity	Negative	Non-smoker	Female	Stage IVA T2N2bM0	006
Oral cavity	Negative	15 Pack-years	Female	Stage III T2N1M0	015
Oral cavity	Negative	Non-smoker	Female	Stage III T3NXM0	023

**Table 2:** Patient treatments

Genome ID	Anatomic site	Treatment Type	Chemotherapy	Radiation Therapy
HNSCC-001	Oropharynx	definitive	cisplatin	Yes
HNSCC-007	Oropharynx	post-operative	cisplatin	Yes
HNSCC-008^a^	Oropharynx	Surgery only	none	No
HNSCC-014	Oropharynx	post-operative	cisplatin	Yes
HNSCC-017	Oropharynx	post-operative	cisplatin	Yes
HNSCC-018	Oropharynx	post-operative	None	Yes
HNSCC-021	Oropharynx	post-operative	cisplatin	Yes
HNSCC-005^b^	Hypopharynx	Surgery only	None	No
HNSCC-003	Larynx	post-operative	None	Yes
HNSCC-004	Larynx	definitive	cisplatin	Yes
HNSCC-009	Larynx	definitive	None	Yes
HNSCC-011	Larynx	post-operative	cisplatin	Yes
HNSCC-019	Larynx	neoadjuvant	abraxane/5FU/cisplatin; cisplatin with RT	Yes
HNSCC-013	Larynx	post-operative	cisplatin	Yes
HNSCC-002	Oral Cavity	post-operative	taxol/cetuximab	Yes
HNSCC-010	Oral Cavity	post-operative	cetuximab	Yes
HNSCC-012^c^	Oral Cavity	Surgery only	None	No
HNSCC-016^d^	Oral Cavity	post-operative	cisplatin	Yes
HNSCC-020	Oral Cavity	post-operative	cisplatin	Yes
HNSCC-024	Oral Cavity	post-operative	cisplatin	Yes
HNSCC-006	Oral Cavity	post-operative	None	Yes
HNSCC-015	Oral Cavity	post-operative	cisplatin	Yes
HNSCC-023	Oral Cavity	post-operative	cetuximab	Yes

^a^no adjuvant treatment given. ^b^2nd primary for H&N. ^c^developed lung metastasis before adjuvant treatment could start. ^d^patient stopped treatment early.

### Sequencing data and bioinformatics workflow

**Figure 1:**
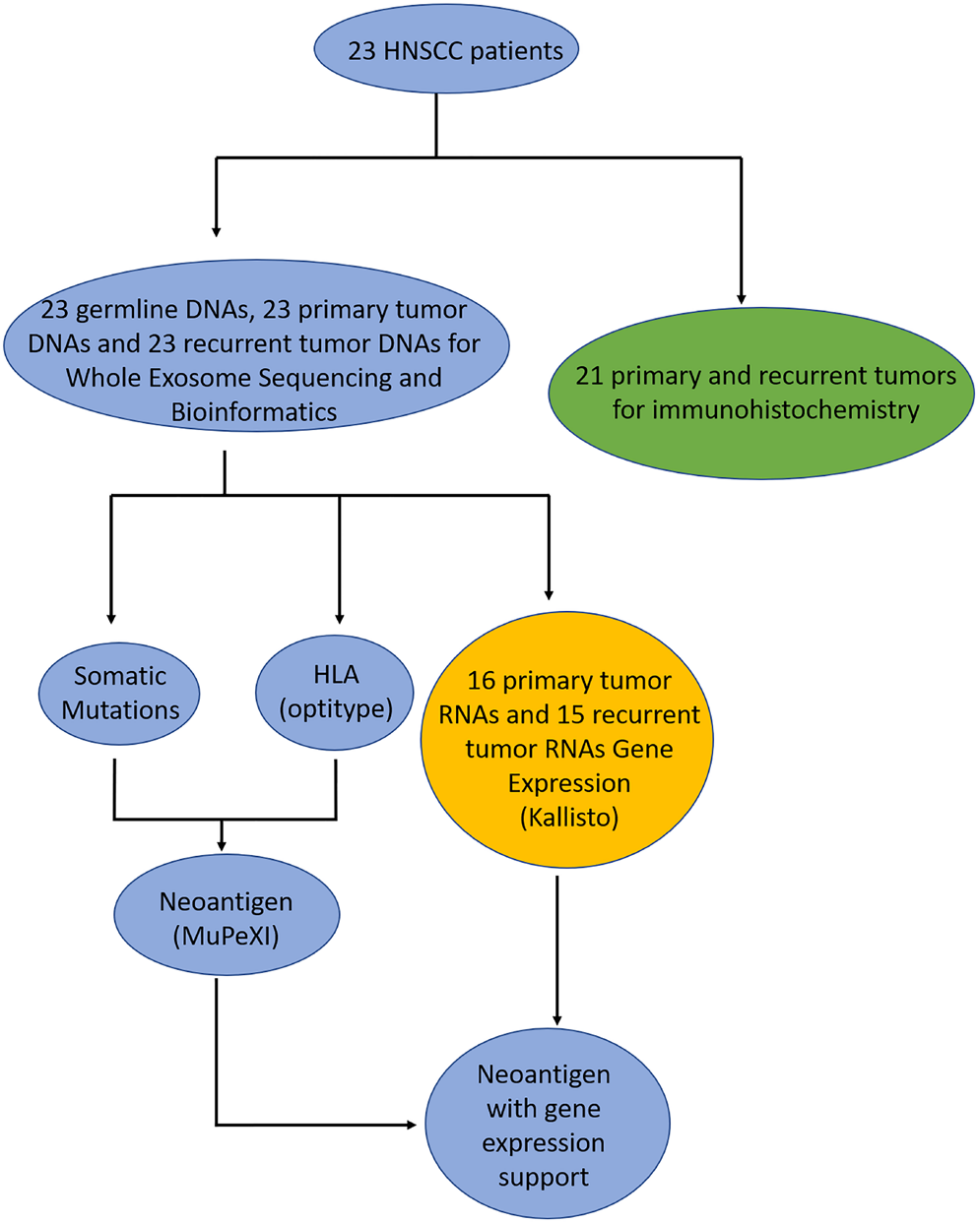
Flowchart of patient samples. The blue circles are the samples where DNA was sequenced and analyzed, green circles are the samples where immunohistochemistry was performed, and yellow are the samples where had RNA sequenced and analyzed.

Of the 23 patients, we sequenced 23 blood samples for germline WES data and 46 paired primary and recurrent/metastatic samples from paraffin blocks to generate WES data, and performed RNA-Seq successfully for 31 samples (Figure 1 and Methods). Next, we generated clean data for downstream analysis based on a standard pipeline. To call high confidant mutations from 46 paired tumor-germline WES data, we used four somatic mutation tools to call mutations and filtered false-positive mutations via bam-readcount tool. Of the RNA-Seq data, for 16 primary tumors and 15 recurrent/metastatic tumors gene expression was predicted using kallisto [26] (Figure 1). To predict neoantigens, we utilized OptiType [27] and MuPeXI [28] to define candidate neoantigens for 46 tumor samples.

### Comparison of somatic mutations between primary and recurrent/metastatic

**Figure 2:**
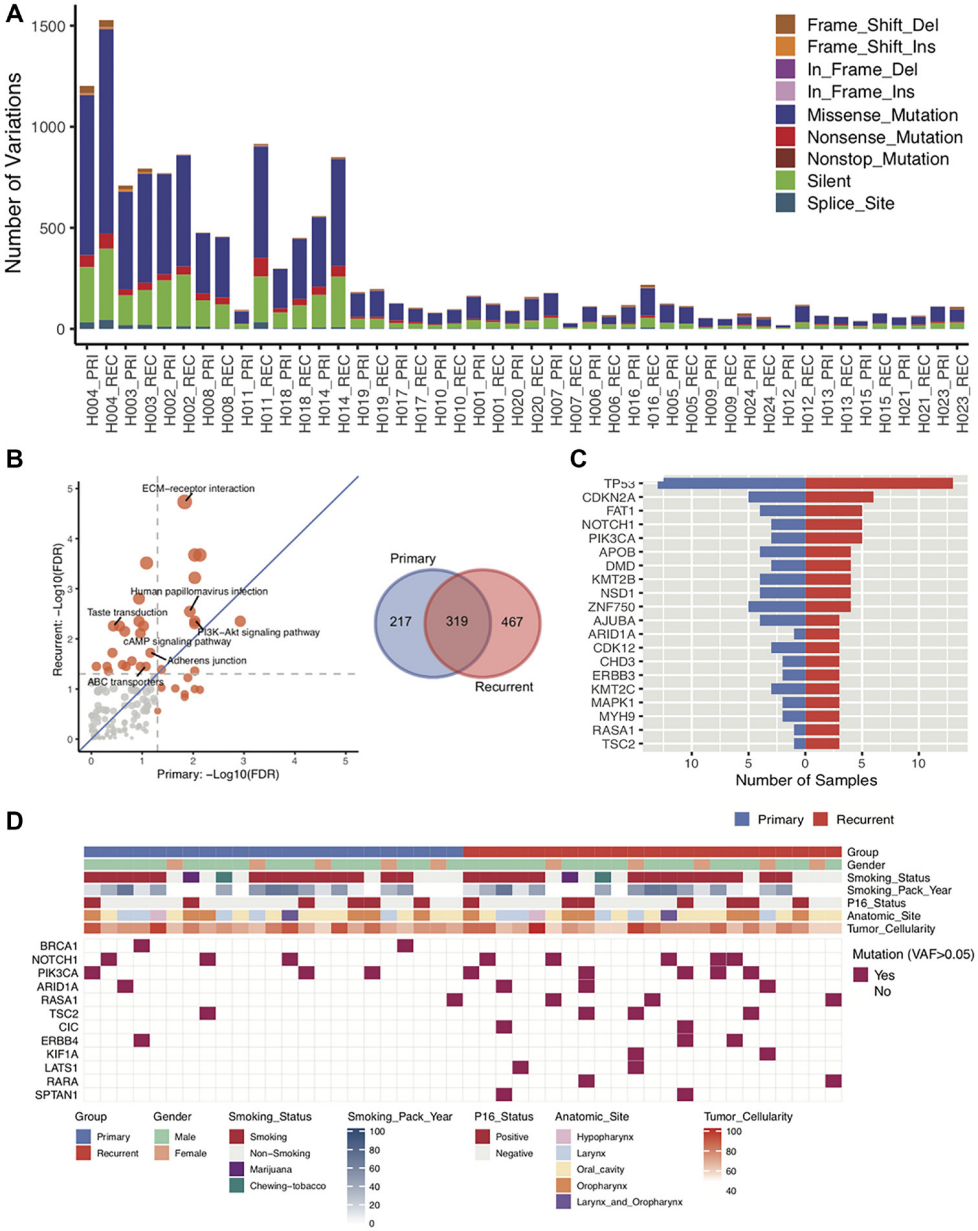
Characterization of DNA sequences of primary and recurrent/metastatic tumors. (**A**) Based on DNA sequencing of 23 paired primary and recurrent/metastatic tumors, the total number of somatic mutations for each patient primary and recurrent/metastatic tumor. The different mutation types are indicated in different colors. (**B**) Based on the sequencing from all 23 paired primary and recurrent/metastatic tumors, KEGG pathways was used to determine which pathways are enriched for mutations in either the primary or the recurrent/metastatic tumor. The Venn Diagram is of the total number of recurrent mutations in the primary tumor alone, recurrent/metastatic tumor alone, or shared. (**C**) The number of patients with mutations in the top 20 most frequently mutated driver genes in both the primary and the recurrent/metastatic tumors. (**D**) Distribution of mutations in driver genes. In both the primary and recurrent/metastatic tumor, the variant allele frequency is greater than 0.5 is indicated for each patient.

Based on the WES, total somatic mutations were identified for each of the patients. Patients were sorted by patients with the highest neoantigen burden. Patients H004, H003, H002, H008, H011, H018, and H014 have the greatest number of total somatic mutations compared with other patients (Figure 2A). Interestingly, three of the patients have a primary tumor in the larynx (Patients H004, H003, and H011) and 3 patients (H008, H018, and H014) have a primary tumor in the oropharynx. There is a general trend that the recurrent/metastatic tumor has more mutations than the primary tumor (Figure 2A). The majority of all somatic mutations are missense mutations and silent mutations. To understand the recurrent mutation effect between primary and recurrent/metastatic tumors, we extract recurrently mutated genes (>1 sample mutated gene) from primary and recurrent/metastatic samples, separately. We detected 536 and 786 recurrently mutated genes from primary and recurrent/metastatic samples, respectively (Figure 2B). Of them, 319 genes shared both of primary and recurrent/metastatic tumors.

We next performed KEGG pathways (http://www.webgestalt.org) analysis to determine if somatic mutations were in pathways related to metastasis. Of them, most of the significant gene enrichment pathways tend toward the relapse due to the generally higher number of mutations in the recurrent/metastatic compared to the primary samples (Figure 2B). Of them, ABC transporters, Adherens junction, cAMP signaling, and Taste transduction pathways were significant only in recurrent/metastatic samples, and extracellular matrix (ECM)-receptor interaction, Human papillomavirus infection, and PI3K-Akt signaling pathways were significant in both of primary and recurrent/metastatic samples. Notably, ECM-receptor interaction pathway was extremely significant in recurrent/metastatic samples meaning that genes related to this pathway are more highly mutated than other pathway mutations in recurrent/metastatic samples. ECM–receptor interaction signaling pathway plays a crucial role in modulating breast cancer metastases [29, 30]. We then checked driver gene inflection for primary and recurrent/metastatic samples via 299 known driver genes [31]. Figure 2C demonstrates the high frequency of mutations in the top 20 driver genes. *TP53* gene is the highest mutated driver gene in both sample groups. We further identified differential mutations in the driver genes between the primary and recurrent/metastatic tumors. In doing so, we identified that *BRCA1* and *NOTCH1* driver genes are highly mutated in primary samples, and *PIK3CA*, *ARID1A*, *RASA1*, *TSC2*, and *ERBB4* were mutated higher in recurrent/metastatic compared with primary samples. Especially, *CIC*, *KIF1A*, *LATS1*, *RARA*, *SPTAN1* genes only mutated in recurrent/metastatic samples (Figure 2D).

### Immune cell infiltration

**Figure 3:**
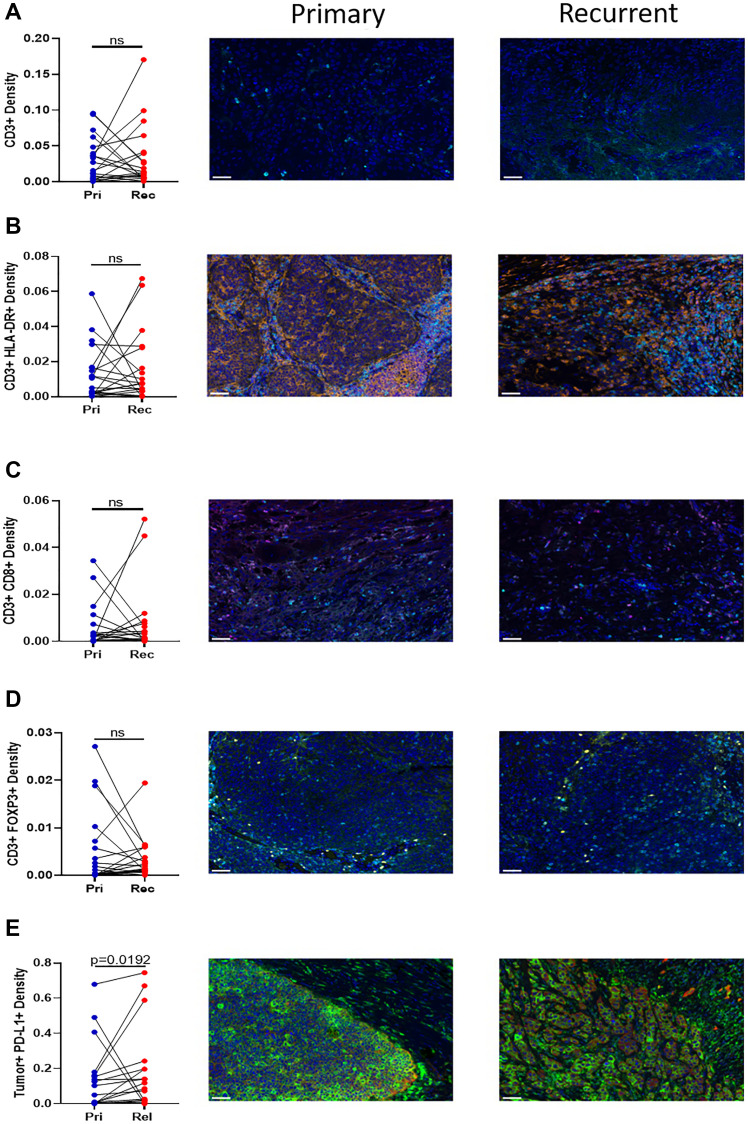
Immunohistochemistry of T cell infiltration into the tumor. Twenty paired blocks from the primary and relapse tumor were stained for (**A**) CD3+ (cyan), (**B**) CD3+ (cyan) HLA-DR+ (orange), (**C**) CD3+ (cyan) CD8+ (magenta), (**D**) CD3+ (cyan) Foxp3+ (yellow), and (**E**) Tumor+ (Pan Cytokeratin red) PD-L1+ (green). All sections contain DAPI (blue). The density of the paired samples are graphed. Significance was determined using a paired *t* test with. A *p*-value is significant if it is less than 0.05, ns = not significant. Images are 20× magnification and the scale bar is 50 μm.

To determine the infiltration of immune cells into the primary and recurrent/metastatic tumors, immunohistochemistry (IHC) was performed. There is no difference in the infiltration of CD3+ cells (Figure 3A), activated T cells (CD3+ HLA-DR+) (Figure 3B), cytotoxic T cells (CD3+ CD8+) (Figure 3C), or CD3+ FOXP3+ cells (Figure 3D) between the primary and recurrent tumor. IHC was also used to determine the surface expression of PD-L1 on tumor cells. There was a significant increase in the tumor expression of PD-L1 between the primary and recurrent/metastatic tumors (Figure 3E).

### Gene expression patterns for immune check point genes

**Figure 4:**
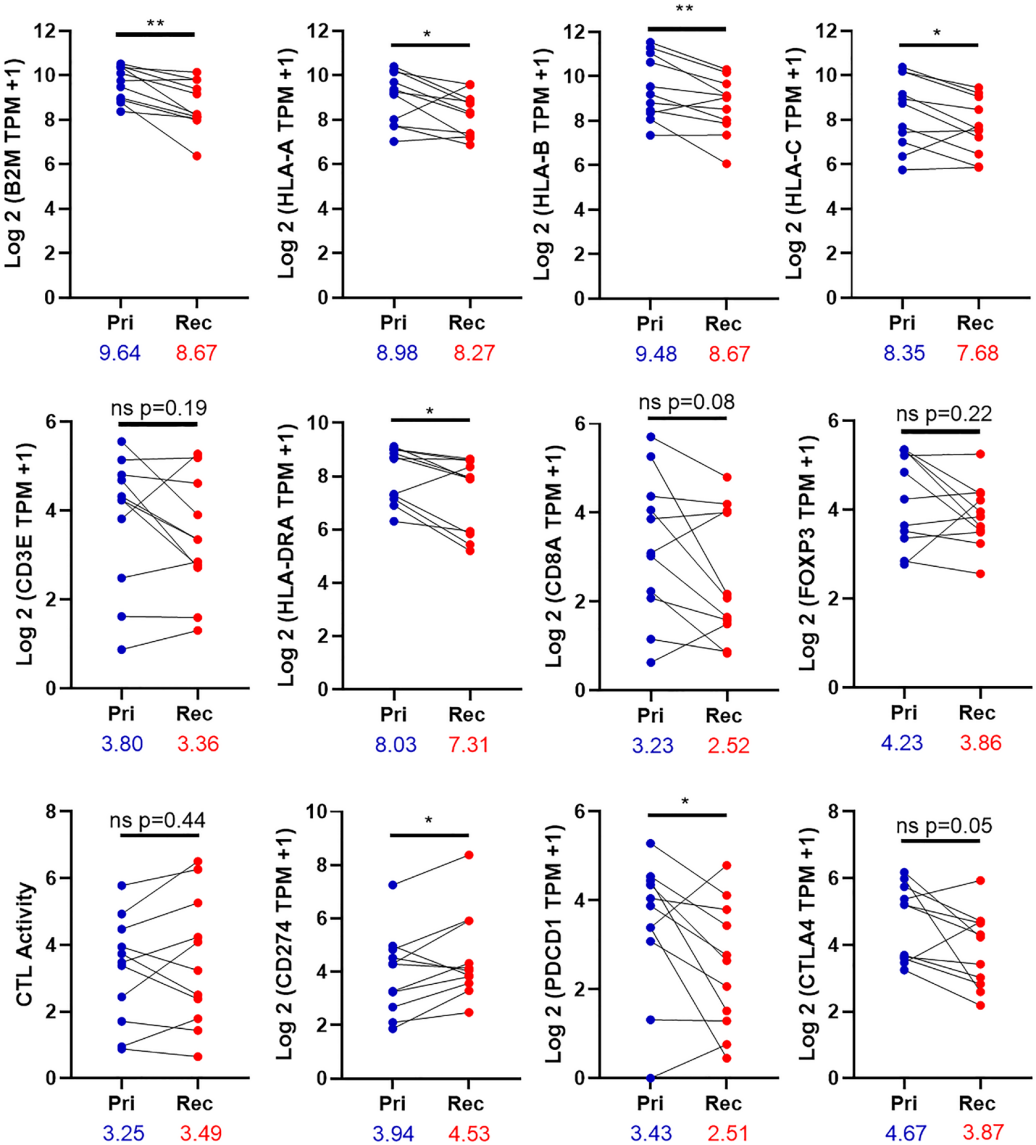
Expression of immune genes in the tumor. The expression of 11 paired primary and relapse tumors was determined by the equation Log 2 (TPM of the indicated gene +1). CTL activity is the geometric mean for the expression of Granzyme A and Perforin. Pri-primary tumor, Rec-Recurrent/metastatic tumor. Significance was determined using a paired *t* test with. A *p*-value is significant if it is less than 0.05. ^*^denoted *p*-value < 0.05 ^**^denotes *p*-value < 0.01, ns = non-significant. The numbers under the X-axis are mean of gene expression.

Eleven pairs of primary and recurrent/metastatic tumors were further determined using Kallisto [26]. There is a significant reduction in the expression of the antigen presenting genes *B2M*, *HLA-A*, *HLA-B*, and *HLA-C* in the recurrent/metastatic tumors relative to the primary tumors (Figure 4). To confirm the immunohistochemistry results, the expression of *CD3E*, *HLA-DRA*, *CD8A*, and *FOXP3* was examined in the primary and recurrent/metastatic tumors. There was a decrease in the expression of all four of these genes in the recurrent/metastatic tumors, relative to the primary tumors. Only *HLA-DRA* was significantly decreased. Cytolytic (CTL) activity is the geometric mean of the expression of perforin and granzyme A [32]. There was no change in the expression of CTL activity between the primary and recurrent/metastatic tumors. The expression of *CD274* (gene for PD-L1) was significantly increased in the recurrent/metastatic tumors relative to the primary tumors, similar to the immunohistochemistry results in Figure 3. Finally, the expression of the checkpoint molecules *PDCD1* (gene for PD-1) and *CTLA4* were examined. There was a decrease in the expression of both molecules, and *PDCD1* was significantly decreased (Figure 4).

### Landscape of HLA genotypes and neoantigens

**Figure 5:**
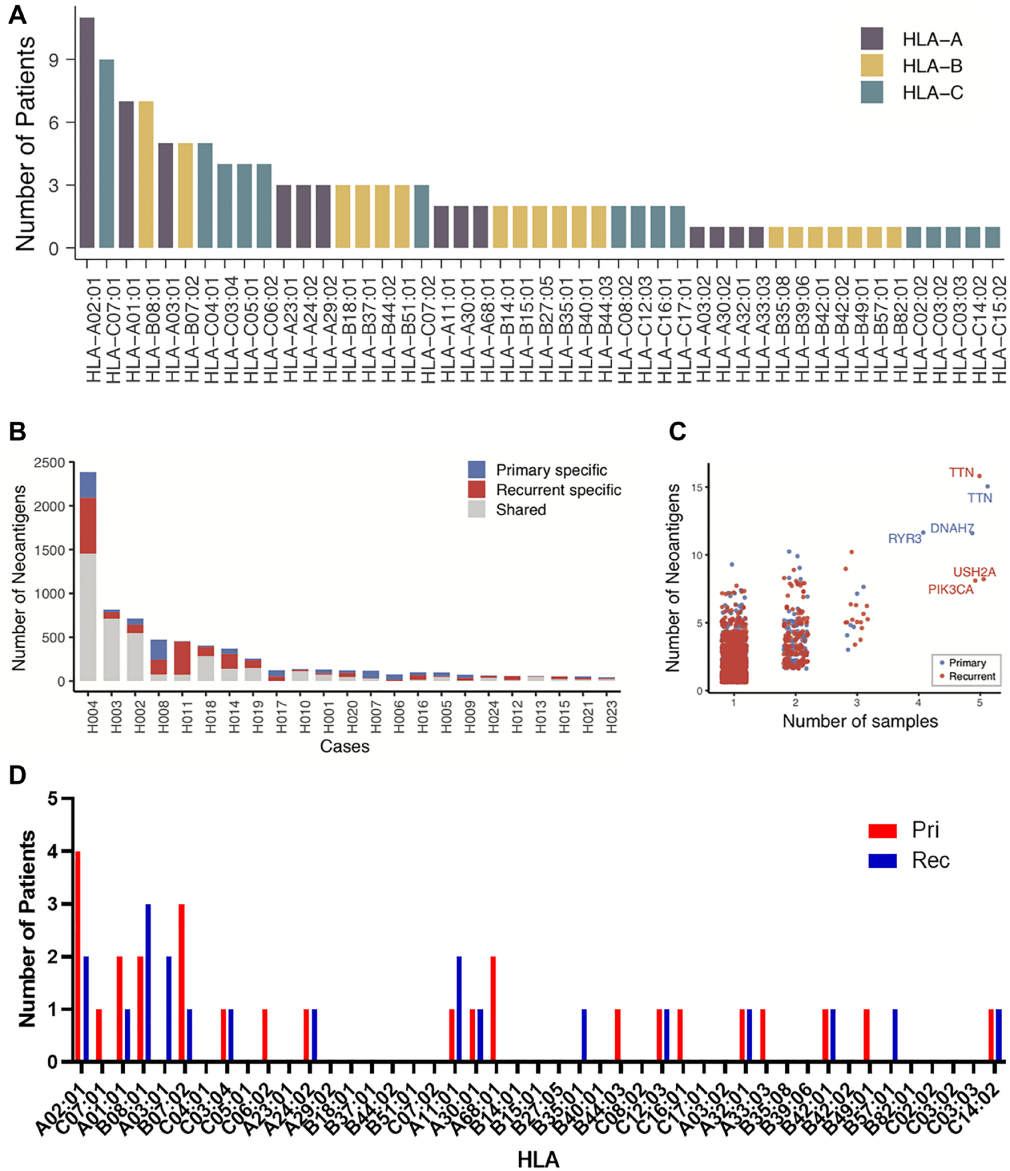
Neoantigen burden and evolution. (**A**) For the 23 patients for which there was DNA, the number of patients expressing HLA-A, -B, and –C alleles in the patient population. (**B**) For 23 patients, the total number of neoantigens predicted in the primary tumor alone (Blue), relapse tumor alone (Red), and shared (Gray). (**C**) The number of predicted neoantigens for each gene was graphed by the number of patients sharing those neoantigens. (**D**) For the patients with neoantigens in shared genes, the number of patients with HLAs predicted to present neoantigens. Neoantigens in primary tumors are in red, neoantigens in recurrent tumors are in blue.

HLA types for each patient were determined with OptiType [27], and the most common HLA type was HLA-A*02:01, which was present in almost half (11/23) of patients (Figure 5A). Other common HLA types include HLA-C*07:01 (9/23 patients), HLA-A*01:01 (7/23), and HLA-B*08:01 (7/23). Using the data from somatic mutations and HLA genotypes, neoantigens were predicted using MuPeXI [28]. Patients H004, H003, H002, H008, H011, H018, and H014 had the highest neoantigen burden (Figure 5B). These are the same patients who had large numbers of somatic mutations in Figure 2A. While there were specific neoantigens in either the primary or the recurrent/metastatic tumor, there was a trend toward more neoantigens in the recurrent/metastatic compared to the primary tumors (Figure 5B). We next sought to determine if genes containing neoantigens were shared between patients. Most neoantigens were unique to an individual tumor. While there were some genes containing neoantigens that were shared between 2 or 3 patients, a total of five genes with neoantigens were shared by 4 or 5 patients (Figure 5C). Of these genes, three (*RYR3*, *DNAH7*, and *TTN)* were identified from the primary tumors. There were three genes (*PIK3CA*, *USH2A, and TTN*) containing neoantigens in the recurrent/metastatic tumors. A summary of the number of predicted neoantigens from each of the identified genes from each patient are listed in Table 3. The amino acid sequence for the predicted neoantigen and the predicted HLA presenting the neoantigen are listed in Supplementary Table 1. It is important to note that while the genes which share the neoantigens are shared between patients, the predicted neoantigen peptides are unique to each patient because the mutations varied between patients. There is one peptide sequence shared between patients. Both Patient 001 and 018 have a predicted neoantigen from 533-542 of PIK3CA in their recurrent tumor. Both patients have a mutation to lysine at position 542.

**Table 3:** Number of neoantigens in selected genes

Anatomic site	Patient ID	RYR3 Primary	DNAH7 Primary	TTN Primary	TNN Recurrent	PIK3CA Recurrent	USH2A Recurrent
Oropharynx	001					1	
Oropharynx	007	9					
Oropharynx	008		3			3	
Oropharynx	014					2	
Oropharynx	017		8				
Oropharynx	018	4		7	6	3	
Oropharynx	021						
Hypopharynx	005						
Larynx	003	5	2		1		3
Larynx	004	9	1	7	11		5
Larynx	009						
Larynx	011						
Larynx	019			5			1
Larynx	013						2
Oral cavity	002		3				
Oral cavity	010						
Oral cavity	012						2
Oral cavity	016			4		2	
Oral cavity	020				4		
Oral cavity	024						
Oral cavity	006						
Oral cavity	015						
Oral cavity	023			3	3		

To determine if the predicted neoantigens were a result of HLAs found in this patient population, we looked at the distribution of the HLAs predicted to present neoantigens from the shared genes (Figure 5D). A total of 14 of the 23 patients are represented in this group. Four patients had neoantigens predicted in primary tumors to be displayed on HLA-A*02:01, and two patients had had neoantigens in the recurrent tumor displayed on this haplotype. Three patients with mutations in recurrent tumors were presented on HLA-B*08:01 and three patients had primary tumor neoantigens predicted to be displayed on HLA-B*07:02. These are on the only other haplotypes utilized by more than 2 patients. This suggests that the presence of neoantigens in these shared genes are not a result of HLA distribution in this population.

### Neoantigens and CTL activation

**Figure 6:**
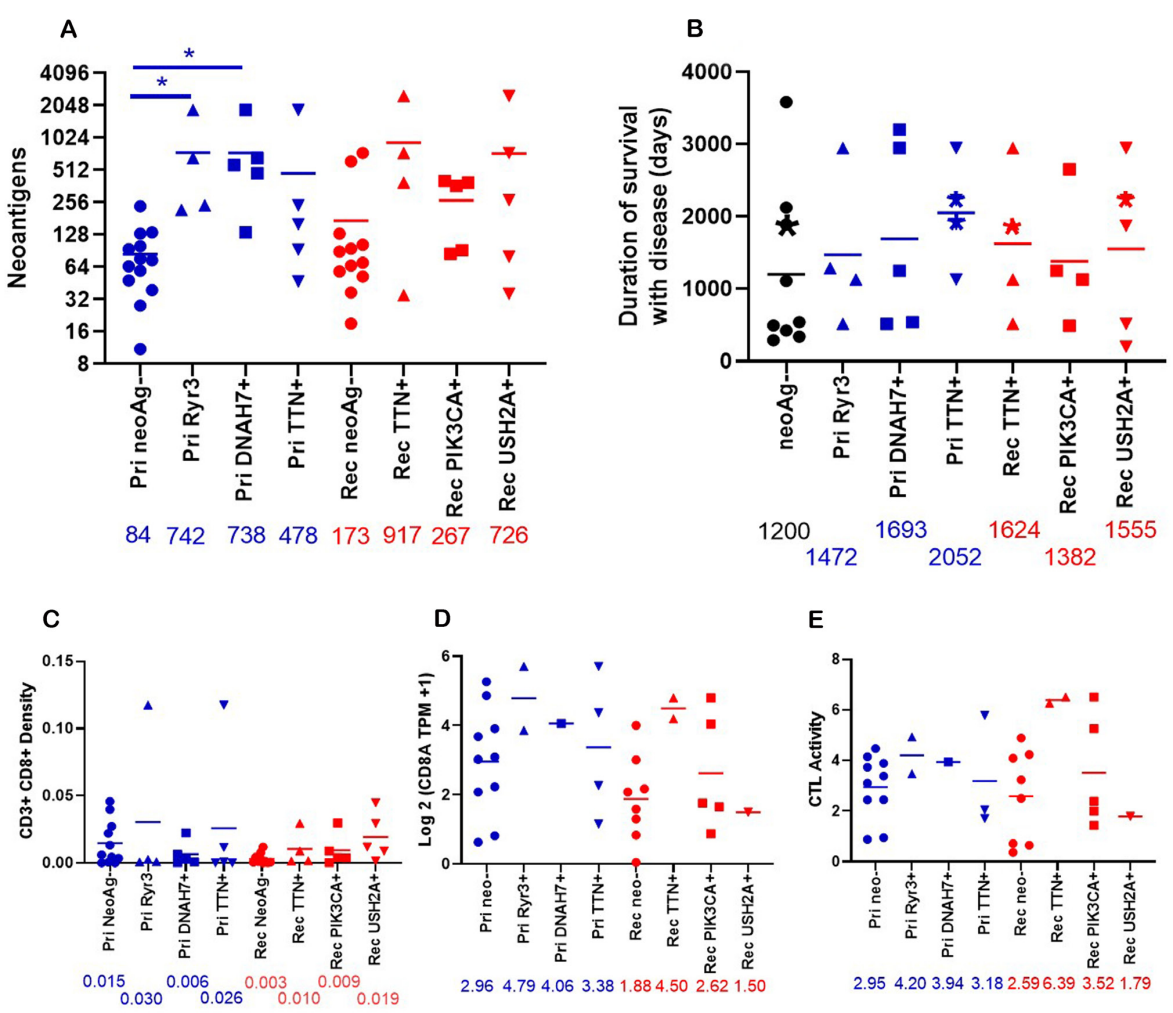
Properties of patients who have neoantigens in shared genes. (**A**) The total number of neoantigens was graphed for primary tumors with neoantigens in Ryr3 (*n* = 4), DNAH7 (*n* = 5), TTN (*n* = 5), or no neoantigens (Pri neoAg-, (*n* = 13)) or relapse tumors with neoantigens in TTN (*n* = 4), PIK3CA (*n* = 5), USH2A (*n* = 5), or no neoantigens (Rel neoAg-, (*n* = 12)). The numbers under the X axis are the mean of neoantigens. ^*^indicates *p* < 0.05. (**B**) The duration of disease for patients with predicted neoantigens in the Primary tumor (Ryr3 (*n* = 4), DNAH7 (*n* = 5), TTN (*n* = 4)) and relapse tumor (TTN (*n* = 4), PIK3CA (*n* = 4), and USH2A (*n* = 5)), or no predicted neoantigens in these genes (neoAg-, (*n* = 9)). Patients with neoantigens in multiple genes are placed in all neoantigens. The asterisks indicate patients who were alive as of writing. (**C**) The density of CD3+ CD8+ cells in the tumor graphed by presence of neoantigens in shared genes. Primary RYR3 (*n* = 4), Primary DNAH7 (*n* = 5), Primary TTN (*n* = 5), Primary no neoantigens (*n* = 11), relapse TTN (*n* = 4), relapse PIK3CA (*n* = 5), relapse USH2A (*n* = 5) relapse no neoantigens (*n* = 10)). Numbers under the X axis are the mean of the density. (**D**) The Log2 (TPM +1) expression of CD8A was graphed by the presence of neoantigens in shared genes. Pri NeoAg- (*n* = 10), Pri Ryr3+ (*n* = 2), Pri DNAH7+ (*n* = 1), Pri TTN+ (*n* = 4), Rel neoAg- (*n* = 8), Rel TTN+ (*n* = 2), Rel PIK3CA+ (*n* = 5), Rel USH2A+ (*n* = 1). The number under the X axis is the mean for each column. (**E**) The CTL activity was graphed by neoantigen status. CTL activity was calculated as described in Figure 4. Pri NeoAg- (*n* = 10), Pri Ryr3+ (*n* = 2), Pri DNAH7+ (*n* = 1), Pri TTN+ (*n* = 3), Rel neoAg- (*n* = 8), Rel TTN+ (*n* = 2), Rel PIK3CA+ (*n* = 5), Rel USH2A+ (*n* = 1). The number under the X axis is the mean for each column.

We sought to determine the effect of these neoantigens in shared genes on the patient. Patients with neoantigens in these shared genes tend toward higher overall neoantigen burden compared to those without neoantigens in these shared genes (Figure 6A). In primary tumors, patients with neoantigens in *RYR3* and *DNAH7* have significantly more total neoantigens compared to patients without neoantigens in these shared genes. The duration of survival with disease was also increased in patients with these neoantigens in shared genes compared to those without (Figure 6B). Without neoantigens in shared genes, the mean duration of survival with disease is 1,200 days. There was a non-significant increase in the patients with neoantigens in shared genes ranging from 1,382–2,052 days. This increase in the duration of survival with disease may be related to higher infiltration of CD3+ CD8+ cells as determined by IHC (Figure 6C). In the primary tumor, there are no significant changes in CD3+ CD8+ density. In the recurrent/metastatic tumors, patients with neoantigens in *TTN*, *PIK3CA*, and *USH2A* increased CD3+ CD8+ infiltration approximately 3-fold compared to patients without neoantigens in these genes. The expression of *CD8A* was increased in primary tumors with neoantigens in *RYR3* and *DNAH7* and in recurrent/metastatic tumors with neoantigens in *TTN* and *PIK3CA* (Figure 6D). CTL activity trended to be increased in patients with the neoantigens in *RYR3* and *DNAH7* in primary tumors and neoantigens in *TTN* and *PIK3CA* in recurrent/metastatic tumors (Figure 6E). This is notable given there was no change in CTL activity in between primary and recurrent/metastatic tumors (Figure 4), but the sample size is small.

## DISCUSSION

In this study, we sequenced the primary and the recurrent/metastatic tumor from 23 HNSCC patients and found an expected increase in the number of mutations in the recurrent/metastatic tumors compared to the primary tumors. By IHC, we found no differences in the infiltration of immune cells, however the recurrent/metastatic tumors had decreased expression of the antigen presenting genes, *B2M*, *HLA-A*, *HLA-B*, *HLA-C*, and *HLA-DR*, and the checkpoint molecule *PDCD1* and increased *CD274* expression compared to the primary tumors. Most importantly, we identified neoantigens in the recurrent genes in four-five patients. These patients have increased total neoantigens, and a trend toward increased duration of survival with disease, infiltration of CD8 cells, and CTL activity. This suggests HNSCC neoantigens can stimulate an anti-tumor immune response.

For the primary and recurrent/metastatic HNSCC tumors, there is an increased mutational burden. It is interesting that the number of mutations is not simply increasing from the primary to the recurrent/metastatic tumor, but they are changing, with the primary and recurrent/metastatic tumors having unique sets of mutations. While the change in mutational burden will have implications for the neoantigen burden, the alteration of mutational burden will have impacts on the biology of the tumor and on tumor metastasis. For example, the upregulation of the PI3K can upregulate matrix metalloproteins and upregulation of extracellular matrix-receptor pathways are associated with metastasis and invasion [21, 22, 24].

Next, we examined the infiltration of T cells into the tumor with immunohistochemistry and gene expression. The expression of MHC I genes (*B2M*, *HLA-A*, *HLA-B*, and *HLA-C*) were decreased significantly. This suggests the ability to present antigens to the infiltrating immune cells is decreased in the recurrent/metastatic compared to the primary tumor. Despite this decrease in MHC I gene expression, this does not correspond to changes in the T cell infiltration. By IHC, there were no significant changes in the infiltration of any examined T cell population in primary and recurrent/metastatic tumors. This is consistent with the gene expression of *CD3E* which also is unchanged between the primary and recurrent/metastatic tumor. While the expression of HLA-DR on T cells is a marker of activation [33, 34], the expression of *HLA-DRA* is significantly reduced. HLA-DR is expressed from other immune cells (such as antigen presenting cells), suggesting the decreased expression of the total tumor is due to the downregulation of *HLA-DRA* in other cells. Interestingly, down-regulation of HLA-DRA is also associated with non-activated antigen presenting cells.

Beyond the infiltration of the cells, CTL activity (geometric mean of perforin and granzyme A expression) is not noticeably different between the primary and the recurrent/metastatic tumors. So, there is no change in either the infiltration of CD3+ CD8+ cells or cytolytic activity. Lastly, by immunohistochemistry, CD3+ FOXP3+ cells and by RNA-Seq, FOXP3 expression is not significantly changed between the primary and recurrent/metastatic tumor. This suggests that the infiltration of T cells in general and more specifically cytotoxic T cells and Tregs are not different between the primary and recurrent/metastatic tumors. However, we did not examine other immune cell populations. More work is needed to further determine the different immune populations within the tumor. It would be interesting to determine the infiltration of other immune cell populations and approximate the activation state of these cells, but this would require a larger data set.

We also tested the expression of check point molecules by IHC and gene expression. PD-L1 is significantly increased on tumor cells by IHC. This increase is reflected by significantly increased expression of *CD274* in the tumor. Both *PDCD1* (gene for PD-1) and *CTLA4* are decreased in the recurrent/metastatic tumor compared to the primary tumor. As has been previously described, the expression of check point molecules, in particular PD-L1, is important for determining the efficacy of check point inhibition therapy [7, 8]. The observation that the expression of these checkpoint molecules changes between the primary and recurrent/metastatic tumors has potential implications for therapeutic development. While additional studies are needed, these results suggest that changes to check point molecule expression may facilitate the relapse of HNSCC.

It is not surprising that the patients with highest numbers of total somatic mutations have the highest neoantigen burden. As with somatic mutations, many neoantigens are shared between the primary and recurrent/metastatic tumor. However, there is a shifting neoantigen burden as there are unique neoantigens in primary tumors and different unique neoantigens in the recurrent/metastatic tumors. Interestingly, there are neoantigens in genes shared between up to five of the 23 patients. The patients which have these neoantigens in shared genes are patients which have higher total numbers of neoantigens. As such, it is not clear if the differences identified are due to the specific neoantigens or to the increased total number of neoantigens. What is clear is that patients with neoantigens in these shared genes also tend to have increased duration of survival with disease. The increased survival may not have been statistically significant, but the increased survival for more than 100 days would be noticeable for the life of the patient. While much more work is needed to expand on these results, the observation that five patients in this relatively small sample have neoantigens in the shared genes is remarkable. The increase in neoantigens and duration of survival with disease tends to be associated with increased CD3+ CD8+ density in the tumor and *CD8A* expression. More interestingly, there is a trend toward increased in CTL activity in the patients with shared neoantigens. This suggests that patients with these shared neoantigens are associated with increased CD8+ T cell infiltration and increased cytotoxic activity, which extends the patient’s life. This raises the possibility that the presentation of certain neoantigens are important for control of tumor growth. This small exploratory study will provide the justification for a larger study of neoantigens in HNSCC.

## MATERIALS AND METHODS

### Patient identification

The HNSCC tumor bank at Washington University was queried for patients that had consented for genomic analysis IRB#201102323. To be included in this study, tumor material had to be available from germline (white blood cells), primary tumors and first recurrence/metastases.

### Sequenced data set and filtering

Of the 23 cases, a total of 69 samples (23 blood samples (for germline), 23 primary tumors, and 23 recurrent/metastatic tumors) had DNA and total RNA independently extracted, whole exome sequencing (WES), and RNA-Seq (performed using NovaSeq 6000 sequencing system). Of the raw data, the WES data included 23 blood normal samples, 23 primary tumors, and 23 recurrent/metastatic tumor samples. RNA-Seq raw data was available for 16 primary tumors and 15 recurrent/metastatic tumors. After that, adapter and low-quality sequences were trimmed from raw 2 × 150 bp paired-end reads using Trim Galore (v0.5.0) (https://www.bioinformatics.babraham.ac.uk/projects/trim_galore). The resulting WES reads were then filtered for the duplicated reads and converted to bam format for use in downstream analysis. For the RNA-Seq, we used trimmed reads as the input to downstream analysis.

### Somatic variant calling

Somatic mutations were determined using an in-house pipeline (https://github.com/ding-lab/somaticwrapper) named as SomaticWrapper, which uses several somatic variant calling tools including Strelka (v2.9.2) [35], Mutect (v1.1.7) [36], VarScan (v2.3.8) [37] and Pindel (v0.2.5) [38]. To generate high confidence mutation callings, we used the mutations that supported at least 2 callers, cutoffs of at least 14 total reads in the tumor and at least 8 in the normal. The mutation list was further filtered by removing variant alleles observed in fewer than 4 reads, present at a variant allele frequency (VAF) less than 0.05 in tumor or higher than 0.01 VAF in the normal. For the candidate somatic mutations, we further filtered low quality mutations by bam-readcount (-q 10 -b 20) (https://github.com/genome/bam-readcount).

### Bulk RNA-seq methods

Transcript quantification was performed using kallisto (v0.44.0) [26], against the GENCODE transcript reference (release 29, GRCh38). Subsequent analysis was performed using R (v3.6.0) and R package ‘tximport’ (v1.12.0) [39] was used to import and aggregate transcript level data to the gene-level. From the transcripts per million (TPMs), gene expression was calculated by log 2 (TPM +1). Significance was determined using Prism Graphpad (V8.4.3) using a paired two-tailed *t* test with values determined to be significant when *p*-value < 0.05.

### Neoantigen prediction

HLA class I genotype was predicted from the WES data using OptiType (v1.2.1) [27]. Combined with HLA genotypes, non-synonymous mutations, and gene expression profile, MHC class I specific binding neoantigens were predicted using MuPeXI (v1.2.0) [28]. The binding affinity of MHC class I with candidate peptides were evaluated with netMHCpan-4.0 [40]. Candidate neoantigens were identified as those with a predicted mutant peptide binding affinity of < 500 nM for peptides of length 8–11 amino acids. After that, the low gene expression (TPM < 0.5) neoantigens were filtered, if the sample has RNA-Seq data. In addition, the inner-duplicated short peptides and the peptides with the same MHCaffinity values between normal and the tumor columns were removed from each sample.

### Quantitative multiplex immunofluorescence

Six-marker multiplex immunofluorescence on 21 formalin-fixed paraffin embedded primary and recurrent/metastatic tumors was performed using the PerkinElmer/Akoya (Marlborough, MA) Opal reagents for multiplex immunofluorescence with the following primary antibodies CD3 (clone LN10, Leica #NCL-L-CD3-565), HLA-DR (clone LN-3, Abcam #ab166777), CD8 (clone 4B11, Leica #PA0183), FoxP3 (clone 236A-37, Abcam #ab20034), panCK (clone PCK-26, Abcam #ab6401), and PD-L1 (clone SP142, Abcam #ab228462), followed by image acquisition on the PerkinElmer/Akoya Vectra 3 automated multispectral microscope and data processing and analysis using the PerkinElmer/Akoya inForm analysis software. The density of CD3+, CD3+ HLA-DR+, CD3+ CD8+ CD3+ FOXP3+, and Tumor+ PD-L1+ cells inside the primary and recurrent/metastatic tumors was determined from the software and was averaged for the primary and recurrent tumors. Significance was determined using Prism Graphpad using a Wilcoxon matched-pairs signed rank test with values determined to be significant when *p*-value < 0.05.

### Statistics

All other comparisons between more than two groups was done using Kruskal-Wallis test followed by Dunn’s multiple comparison’s test. Differences were determined to be significant when *p*-value < 0.05.

## SUPPLEMENTARY MATERIALS





## Abbreviations

ECM: Extracellular Matrix
HNSCC: Head and neck cell squamous-cell carcinomas
HPV: human papilloma virus
IHC: immunohistochemistry
WES: whole exome sequencing

## Notes

**Editorial note**

This paper has been accepted based in part on peer-review conducted by another journal and the authors' response and revisions as well as expedited peer-review in Oncotarget.
