# Supplementary material for: Genomic and neoantigen evolution from primary tumor to first metastases in head and neck squamous cell carcinoma
Source: Oncotarget. 2021 Mar 16;12(6):534–48. doi: 10.18632/oncotarget.27907 (PMC7984826; doi:10.18632/oncotarget.27907)
Supplement: Supplementary file 1 [file oncotarget-12-534-s001.pdf]

Genomic and neoantigen evolution from primary tumor to first metastases in head and neck squamous cell carcinoma

SUPPLEMENTARY MATERIALS

Supplementary Table 1: List of predicted neoantigen peptide sequences and presenting HLAs for all shared neoantigens. See Supplementary Table 1
