# Supplementary material for: Genomic and neoantigen evolution from primary tumor to first metastases in head and neck squamous cell carcinoma
Source: Oncotarget. 2021 Mar 16;12(6):534–48. doi: 10.18632/oncotarget.27907 (PMC7984826; doi:10.18632/oncotarget.27907)
Supplement: Supplementary file 2 [file oncotarget-12-534-s002.docx]

**Supplementary Table 1: List of predicted neoantigen peptide sequences and presenting HLAs for all shared neoantigens**

| RYR3 Primary | | |
| --- | --- | --- |
| Patient ID | HLA haplotype (peptide #) | Core Peptide |
| 003 | A02:01 (2)  B08:01 (2)  B49:01 (1) | 4218-GLVEWAKNIRV-4228  3084-TPRERSIPGM-3093, 4222-WAKNIRVTKI-4231  4220-VEWAKNIRV-4228 |
| 004 | A30:01 (1)  B08:01 (7)  B42:01 (1) | 2330-VGILSIPLK-2338  621-DNLLLRRNLLL-630  2324-VPTEDLVGIL-2333 |
| 007 | A33:03 (3)  A68:01 (3)  B07:02 (2) | 770-NTDGLFFPVMRFSAGVKVR-788  770-NTDGLFFPVMR-780  776-FPVMRFSAGV-785 |
| 018 | A11:01 (3)  B07:02 (1) | 427-RTAAPITLPIK-437  434-LPIKEVLQTL-443 |

| DNAH7 Primary | | |
| --- | --- | --- |
| Patient ID | HLA haplotype (peptide #) | Core Peptide |
| 002 | A02:01 (3) | 1048-ELLELILKELNEYL-1061 |
| 003 | A02:01 (2) | 2675-ILESALAAIDTLTA-2688 |
| 004 | C03:04 (1) | 1715-LMSGDIIQM-1723 |
| 008 | A01:01 (1)  A02:01 (1)  C06:02 (1) | 2743-LGDMRFLQSLY-2753  2751-SLYEYDKDNI-2760  2746-MRFLQSLYEY-2755 |
| 017 | A02:01 (5)  B07:02 (1)  C07:02 (1)  C12:03 (1) | 1537-LPKFLSHDLALFEGITSDLF-1556  1537-LPKFLSHDLAL-1547  1539-KFLSHDLAL-1547  1540-FLSHDLALF-1548 |

| TTN Primary | | |
| --- | --- | --- |
| Patient ID | HLA haplotype (peptide #) | Core Peptide |
| 004 | A30:01 (5)  B42:01 (1)  C03:04 (1) | 32861-ALREAAVVYKPAVSTK-32876,  10740-KVLKKAVTEEK-10750,  21002-KPGIPNGPIK-21011  21002-KPGIPNGPI-21010  32865-AAVVYKPAV-32873 |
| 016 | A32:01 (2)  B44:03 (1)  C16:01 (1) | 22498-KCVLSWLPPL-22507  22496-SEKCVLSWL-22503  22499-CVLSWLPPL-22507 |
| 018 | A11:01 (3)  A24:02 (4) | 959-SVTLECHISR-968, 9502-QEIDIMELLK-9511  966-ISRYPSPTVTWY-977 |
| 019 | A68:01 (2)  C14:02 (3) | 4980-DGIPLVASKK-4989  4976-KWYKDGIPLV-4985 |
| 023 | A01:01 (1)  B08:01 (2) | 33445-MMETRESLSSY-33455  33441-QMMETRESL-33452 |

| TTN recurrent | | |
| --- | --- | --- |
| Patient ID | HLA haplotype (peptide #) | Core Peptide |
| 003 | A02:01 (1) | 25866-YIFEKRDKEGV-25873 |
| 004 | A30:01 (10)  C03:04 (1) | 32861-ALREAAVVYKPAVSTK-32876,  5406-RVTLREPTSFIKK-5418,  10740-KVLKKAVTEEK-10750, 27155-STRAYVDTT-27163  32865-AAVVYKPAV-32873 |
| 018 | A11:01 (2)  A24:02 (4) | 959-SVTLECHISR-968  966-ISRYPSPTVTWY-977 |
| 020 | A11:01 (3)  C12:03 (1) | 3591-KINSKVEGHK-3600, 11491-KKVPEAIPPK-11500  3586-SAYLKINSKV-3595 |
| 023 | A01:01 (1)  B02:01 (2) | 33445-MMETRESLSSY-33455  33441-QMMETRESL-33452 |

| PIK3CA recurrent | | |
| --- | --- | --- |
| Patient ID | HLA haplotype (peptide #) | Core Peptide |
| 001 | A03:01 (1) | 533-AISTRDPLSK-542 |
| 008 | A02:01 (2)  B57:01 (1) | 811-MLTLQIICIMENI-823  816-IICIMENIW-824 |
| 014 | B07:02 (2) | 446-WPVSHGLEDLL-456 |
| 016 | A32:01 (2) | 88-RLCDLQLFQPFLKV-101 |
| 018 | A11:01 (3) | 532-KAISTRDPLSK-542 |

| USH2A recurrent | | |
| --- | --- | --- |
| Patient ID | HLA haplotype (peptide #) | Core Peptide |
| 003 | A02:01 (1)  B02:01 (2) | 206-ILVKKCIHL-214  206-ILVKKCIHL-214 |
| 004 | B08:01 (1)  B42:01 (2)  C03:04 (2) | 3972-WAQAKSAHSVL-3982  3970-APWAQAKSAHSVL-3982  1782-LAFTQVDQL-1790, 3974-QAKSAHSVL-3982 |
| 012 | A08:01 (2) | 2302-HSFRVQACMAK-2312 |
| 013 | B35:01 (2) | 52-VPTQAVCGF-60, 60-FPDRSTFCH-68 |
| 019 | C14:02 (1) | 2536-VFPPILLDV-2544 |

For each HLA haplotype, we have indicated the number of predicted peptides. The peptide sequences are listed. The shared peptides are in black, amino acids unique to one peptide in red, and the mutated amino acid is highlighted in yellow.
